# A genomic ruler to assess oncogenic transition between breast tumor and stroma

**DOI:** 10.1371/journal.pone.0205602

**Published:** 2018-10-16

**Authors:** Shubhada Dhage, Amanda Ernlund, Kelly Ruggles, Deborah Axelrod, Russell Berman, Daniel Roses, Robert J. Schneider

**Affiliations:** 1 Department of Surgery, New York University School of Medicine, New York, New York, United States of America; 2 Perlmutter Cancer Center, New York University School of Medicine, New York, New York, United States of America; 3 Department of Microbiology, New York University School of Medicine, New York, New York, United States of America; 4 Department of Medicine, New York University School of Medicine, New York, New York, United States of America; King Faisal Specialist Hospital and Research Center, SAUDI ARABIA

## Abstract

**Background:**

Cancers induce gene expression alterations in stroma surrounding tumors that supports cancer progression. However, it is actually not at all known the extent of altered stromal gene expression enacted by tumors nor the extent to which altered stromal gene expression penetrates the stromal tissue. Presently, post-surgical “tumor-free” stromal tissue is determined to be cancer-free based on solely on morphological normality—a criteria that has not changed in more than 100 years despite the existence of sophisticated gene expression data to the contrary. We therefore investigated the extent to which breast tumors alter stromal gene expression in three dimensions in women undergoing mastectomy with the intent of providing a genomic determination for development of future risk of recurrence criteria, and to inform the need for adjuvant full-breast irradiation.

**Methods and findings:**

Genome-wide gene expression changes were determined in histopathologically normal breast tissue in 33 women undergoing mastectomy for stage II and III primary invasive ductal carcinoma at serial distances in three dimensions from the tumor. Gene expression was determined by genome-wide mRNA analysis and subjected to metagene mRNA characterization. Tumor-like gene expression signatures in stroma were identified that surprisingly transitioned to a plastic, normalizing homeostatic signature with distance from tumor. Stroma closest to tumor displayed a pronounced tumor-like signature enriched in cancer-promoting pathways involved in disruption of basement membrane, cell migration and invasion, WNT signaling and angiogenesis. By 2 cm from tumor in all dimensions, stromal tissues were in transition, displaying homeostatic and tumor suppressing gene activity, while also expressing cancer supporting pathways.

**Conclusions:**

The dynamics of gene expression in the post-tumor breast stroma likely co-determines disease outcome: reversion to normality or transition to transformation in morphologically normal tissue. Our stromal genomic signature may be important for personalizing surgical and adjuvant therapeutic decisions and risk of recurrence.

## Introduction

Surgical resection of the primary tumor and adjuvant therapy are mainstays of local breast cancer treatment to prevent the growth and metastatic spread of breast cancer [1]. After local surgical resection and adjuvant therapy, the risk of breast cancer recurrence varies widely, in part based on stage and grade [2–5], and does not include parameters of tumor-stromal interaction. It is well established that cancers induce gene expression alterations in the stroma surrounding tumors that can support cancer progression [6–8], Adjacent morphologically normal tissue in the breast post-surgery can harbor pre-neoplastic and neoplastic gene expression changes that are undetectable by histopathology, which in concert with a reactive stromal environment, can give rise to cancer recurrence at primary and distant sites [9]. Detecting occult disease through genetic approaches would allow for a more informed understanding and redefinition of a “clean” surgical margin, and might inform personalized targeted adjuvant therapy to genetically aberrant but histopathologically normal stroma based on individual tumor-stromal characteristics.

Studies have shown that breast tumors remodel the surrounding stroma both anatomically and genetically, making it more receptive to cancer cell invasion, metastasis and recurrence [8, 10–16], Although poorly described, the tumor stroma is altered in such a manner as to reprogram gene expression, making it a better host and enabler of future cancer development [8, 10–15]. Two biological processes have been proposed to describe the interaction between the stroma and tumor [10, 17]: tumor-stromal co-evolution [18], and field cancerization [17]. Regardless of mechanism, it is not known whether oncogenic cancer-promotion in stroma is actually a field that penetrates stroma in the absence of morphological transformation of epithelial cells, or just independent areas of altered gene expression surrounding the tumor. Indeed, the dynamic micro-environment of the tumor stroma has been described as either cancer promoting or inhibiting [19]. A genetic signature has been described in morphologically normal tissue apart from tumor, consistent with increased cell proliferation, activating pathways, and the wound healing response [20]. In contrast, other studies found stromal gene signatures associated with pathways that instead oppose cell proliferation in the cancer-adjacent extra-tumoral microenvironment [21–24]. A stromal gene expression profile has also been reportedly associated with poorer outcomes for patients with estrogen receptor (ER) positive breast cancer [21–24].

Conflicting findings may derive from the fact that studies to date have relied on opportunistic biopsies obtained at random distances from the tumor, from which gene expression changes in the stroma were described. We sought to determine if there is a spatial relationship between the tumor and the stromal landscape that could be identified by gene expression transitions between cancerous tissue, cancer promoting tissue and homeostatic normal tissue. We show that spatially reproducible and genetically defined stromal regions exist within cancer-free “normal” tissue that displays a gradient from stromal gene expression profiles corresponding to tumor-like and pro-transforming to tumor-distant tissue, expressing both key pro-transforming and homeostatic normalizing genes.

## Materials and methods

### Patients

Thirty-three patients undergoing mastectomy for first occurrence primary, invasive ductal breast cancer from 2009–2012 were accrued to trial (NYU Langone Health Institutional Review Board approved protocol NYU#09–1695) with signed consent to use their tissues in genetic and genomic studies. All clinical investigation was conducted according to the principles expressed in the Declaration of Helsinki. All patients underwent a mastectomy with lymph node sampling. None of the patients had multi-centric disease or underwent neo-adjuvant therapy. The majority of patients were stage II (57%), and 50% of all tumors were poorly differentiated (S1 and S2 Tables). Patients were distributed evenly with respect to lymph node positivity. Twenty-nine percent of tumors were HER2^+^, and 12% were triple negative (ER/PR/Her2^-^).

#### Tissue and RNA

Bulk tissue was isolated from mastectomy specimens immediately following surgery (within 30 min), initiating at the gross tumor edge, then every 5 mm up to 20 mm in the longest 3-dimensional axis in the breast from the tumor-stromal clean surgical border (devoid of cancer cells) from all patients, determined by pathological examination. Bulk tissue was used because it captures a more complete gene expression profile of both pre-malignant epithelial cells and reactive stromal cells present in the tumor adjacent stroma. Tumor specimens and stroma were sectioned every 5 mm in two directions along the longest axis from the tumor-stromal interface up to 20 mm from the tumor-stromal margin. Twenty mm was the cut-off for serial three-dimensional sampling because it could be obtained from all mastectomy specimens and in some specimens was the last stromal distance that could be sampled without adipose or chest wall tissue. All specimens were pathologist verified as enriched in tumor or stroma. The cancer cell-free stromal tissue was determined to be devoid of cancer cells by pathologist serial sectioning and staining at the tumor margin, as standard practice for determination of cancer-free tumor margin analysis. For stromal tissues, 5 mm sections were used for genome-wide mRNA expression analysis, which corresponds to ~5x10^5^ mixed stromal cells. Therefore, even if there was contamination of each 5 mm section by 1x103 cancer cells (unlikely as they would have been detected by pathological staining), the cancer cell gene expression signal would account for no more than 0.002% of the stromal cell signal, and which his undetectable and would influence the stroma gene expression data obtained. A total of 108 specimens were analyzed using Affymetrix U133A 2+ human gene expression arrays. RNA was purified from specimens by RNeasy chromatography (Qiagen), analyzed for high quality and quantified for RNA integrity by Agilent Bioanalysis.

### Microarray data normalization and bioinformatics analyses

Data were background corrected using the RMA algorithm and sketch quantile normalized using Affymetrix Expression Console. Genes with an inter-quartile range >0.5 were included in the analysis.

#### Gene expression and profile identification

**Fig 1:**
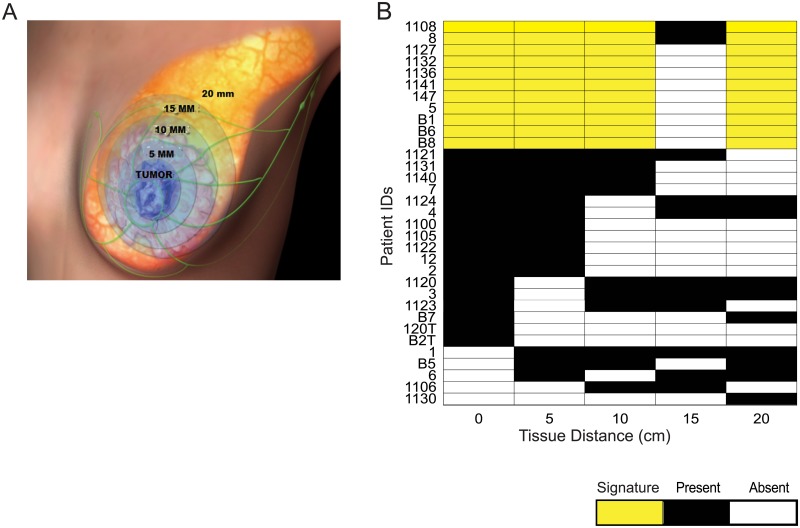
Methodology for tissue sampling. (A) Tissue samples were obtained from tumor (0 mm) and tumor adjacent tissue at 5 mm, 10 mm, 15 mm, and 20 mm away from the pathologic gross tumor edge defined as the margin devoid of cancer cells. (B) Samples from tumor at 5 mm, 10 mm 15 mm, 20 mm collected from each patient. At certain distances specimens could not be obtained due to anatomic considerations (blood vessels, adipose tissue, chest wall). Panel also shows discreet segregation into two groups, tumor (black shading) and non-tumor (white shading). Present indicates the patient had a sample used for data analysis obtained at a particular distance. Absent indicates that tissue at a particular distance was unable to be used for data generation. Tissue samples in yellow were used to derive tumor and tumor-like gene signatures.

Microarrays from patients with tissues represented at tumor (0 mm), 5 mm, 10 mm, and 20 mm (see Fig 1B, samples in yellow) were analyzed for gene expression and differentially expressed genes were identified by comparing specimens for each stromal distance compared to tumor specimens for each patient (n = 11 patients) using the limma package, with paired moderated t-test and utilizing a threshold cut-off of FDR <0.01 and fold-change ≥2 for significance. Three comparisons were made: tumor *vs*. 5 mm, tumor *vs*. 10 mm, tumor *vs*. 20 mm, for patients from all groups. Duplicate sample microarrays within a patient were averaged. The list of differentially regulated genes from each stromal comparison to tumor were then concatenated, yielding a list of 2374 genes. Genes within the concatenated list that were differentially expressed in all stromal distances compared to tumor were considered genes that distinguished the stroma from the tumor, corresponding to 924 probe sets. Genes expressed in common between tumor and stroma were considered to be those that were not in the intersect of differential gene expression across all stromal groups consisting of 1004 genes.

### Molecular and biochemical pathway analysis

Pathway analysis was performed using Ingenuity Pathway Analysis or Go Enrichment Analysis. Tumor-like gene set was analyzed for pathway enrichment by performing a hypergeometric test with Benjamini-Hochberg correction, FDR < 0.01. Network clustering was done using k-means clustering. STRING network analysis was performed utilizing the highest (0.9) confidence minimum required interaction score, showing no more than 10 interactors and removing all disconnected nodes in the network. DAVID pathway analysis of tumor-like genes was also performed by splitting the list of tumor-like genes into upregulated and downregulated and analyzing the top 10 significantly upregulated or downregulated pathways using an enrichment score cut-off > 1.3. IPA analysis was used to analyze pathways in the 20 mm region by analyzing mRNAs uniquely differentially expressed in the 20 mm region alone. Pathway activation was predicted using IPA upstream regulator analysis software, pathways were considered activated or inhibited based on z-score > 2, pathways with unbiased significant *P* < 0.05 were selected.

#### Non-negative matrix factorization

The NMF method was used for multi-dimensional genome-wide mRNA expression analysis. NMF reduces the dimensionality of biological data by capturing genes that tend to be co-expressed across samples, referred to as metagenes, then uses the metagenes to group samples in an unsupervised manner [25, 26]. NMF lends itself to identification of gene expression changes across multiple tissue compartments in multiple dimensions. Rank basis selection and factorization were carried out using the NMF R package. Rank k = 5 was determined to give the most robust clustering based on residual reduction analysis, with 5 metagene clusters used to group patient samples as described [27]. Each metagene cluster was examined for enrichment of each tissue type category (tumor and stromal distances).

#### Trends in gene expression in tumor and stroma

To examine trends in gene expression in tumor and tumor-like signatures, the expression of each gene was averaged across patient specimens for all distances and the average of all genes was examined at each distance of tumor and stroma. Pearson correlation was used to compare the association of each patient stromal specimen to their matched tumor specimen using the tumor-like signature. To identify trends in gene expression for the subset of genes that distinguish only 20 mm tissue (and no other distances of stroma) from tumor, we examined genes that were differentially expressed at 20 mm in stroma compared to tumor, but were not differentially expressed at 5 mm, 10 mm, or 15 mm in stroma compared to tumor. All genes upregulated or downregulated at each distance were averaged to obtain a trend of gene expression across stromal distances.

#### Nanostring validation

Microarray genes were selected from the gene expression profile of the 20 mm region. These genes showed either an upward trend from tumor at 5 mm, 10 mm, 20 mm, or downward trend. Nanostring was performed utilizing the pan-cancer pathway analysis gene set examining 3 patients with paired tumor and stromal samples. 50 ng of purified RNA was isolated from paraffin blocks and hybridized with the Pan Cancer Pathway code set. NSolver software was used to quantify raw counts, to perform quality control, and to normalize data. Genes that overlapped between microarray data and pan-cancer Nanostring data showed concordance in trends in gene expression when stroma was compared to tumor.

#### Data accession

Genome-wide gene expression array data was deposited and can be found at GEO #GSE120129.

## Results

### Genomic categorization of peri-tumoral tissue into two distinct regions using virtual separation methodologies

**Fig 2:**
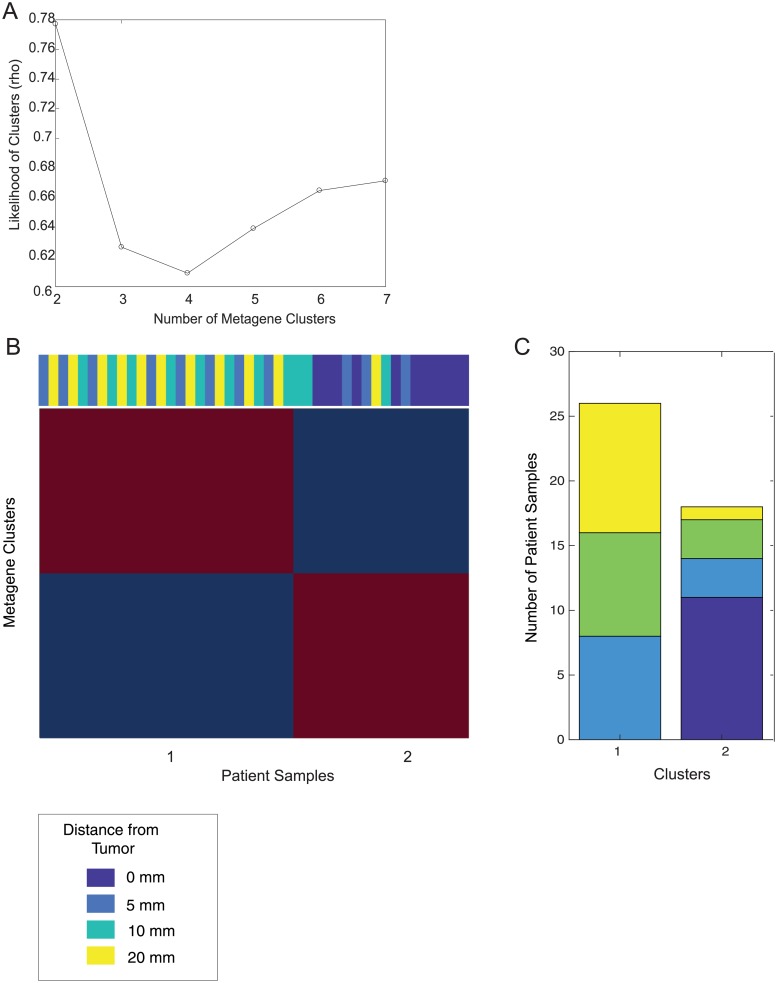
Unsupervised sample clustering using NMF. (A) Dispersal coefficients for sample clustering using 2, 3, 4, 5, 6 and 7 clusters. (B) Heatmap of metagene clusters with patient samples based on distance from tumor in color bar along the top. (C) The number of samples from each distance of tissue present in each metagene cluster.

We investigated gene expression changes in the peri-tumor stromal tissue based on 3-dimensionsal distance from tumor, obtaining specimens from tumors and every 5 mm from stroma initiating at the tumor-stroma border in the longest axis in the breast at mastectomy (Fig 1A). To increase the ability to detect reproducible gene expression patterns across patients, 11 of 33 patients with tissue samples representing almost all distances (0, 5, 10, 20 mm) were segregated for further analysis (Fig 1B). An unsupervised blind source metagene statistical separation methodology was employed across all genes for mRNA expression levels, known as non-negative matrix factorization (NMF), to identify stromal specimen sub-groups, and stromal specimens that segregated into two highly reproducible groups (Fig 2A). The majority of stromal specimens clustered into Group 1. The second cluster, Group 2, primarily showed enrichment of specimens corresponding to tumor samples. Not surprisingly, these data indicate that the tumor and tumor-free stroma up to 20 mm from the tumor-stromal border segregates by gene expression profile in an unsupervised analysis. Interestingly, a small subset of stromal specimens clustered with Group 2. These specimens were from several patients including patient 5 where all samples at 0, 5, 10, and 20 mm all clustered with group 2, patient 8 whose 0 mm and 5 mm specimens clustered with group 2, and patient 147 whose 0 mm and 5 mm specimens clustered with group 2. These patients displayed a very high disease burden in the breast with extensive axillary lymph node involvement (Stage IIIC; Fig 2B and 2C). These findings suggest that stromal gene expression, severe disease burden and extensive lymph node involvement may associate strongly with tumor gene expression despite the absence of cancer cells in stroma. We therefore asked whether histopathologically normal stroma associated with less severe disease burden displays less pronounced but biologically important tumor-like gene expression as well.

### Histologically normal stroma displays a tumor-like gene expression

**Fig 3:**
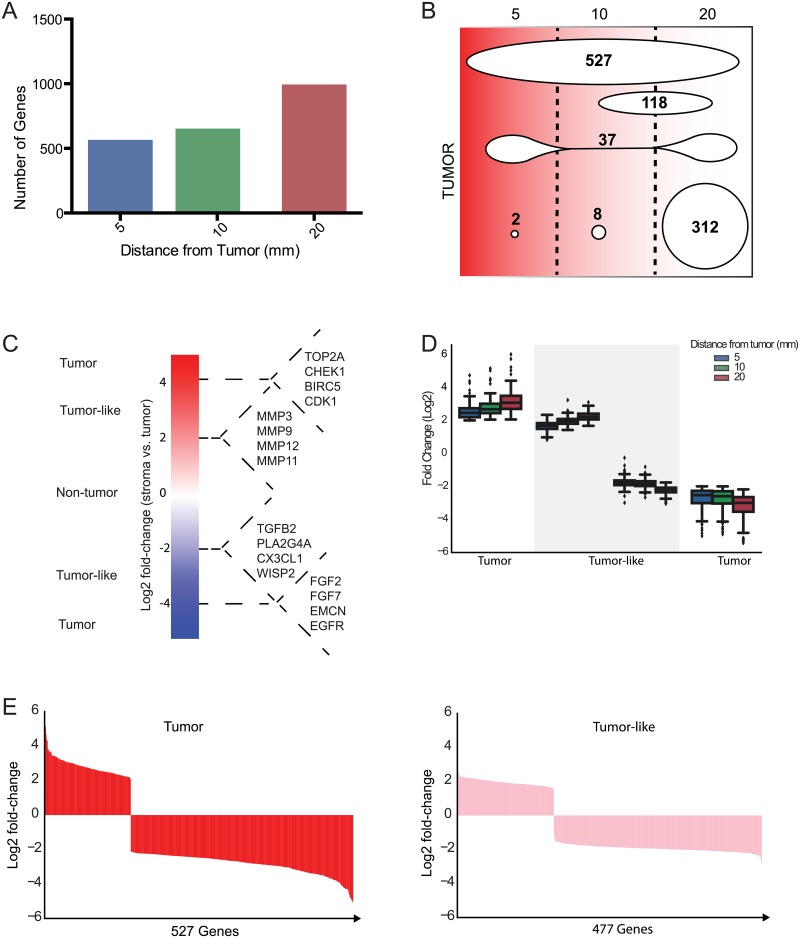
Derivation of a tumor-like stromal gene signature. (A) The number of differentially expressed genes when comparing tumor (0 mm) *vs*. 5 mm, *vs*. 10 mm, and *vs*. 20 mm of cancer-free stroma. (B) Gene lists from 5, 10, and 20 mm of stroma were compared for overlap in gene expression identity. Gene expression in the intersect of all gene lists (527) comprises the tumor gene expression signature, while gene expression in the union of the gene lists excluding the intersect comprise the tumor-like gene expression signature (bottom). (C) Schematic representation of data demonstrating segregation of gene expression as non-tumor, tumor-like, and tumor. (D) Trends in fold-change of tumor and tumor-like gene expression across distances. (E) Log_2_ fold change of entire gene list for tumor or stroma tumor-like genes showing the breadth of change represented by the width of displayed gene change patterns.

To further investigate the finding that some stromal tissue segregates with tumor by displaying similar gene expression to tumor, we employed a supervised strategy. Previous studies have attempted to define altered stroma surrounding the tumor based on common expression of a subset of genes with tumor. However, these studies used biopsy specimens obtained opportunistically at generally random or unknown distances from tumor and therefore could not determine how or whether gene expression changes in relation to distance of stromal sample from tumor. In order to separate house-keeping genes with similar expression profiles across tumor and stroma from genes important for the cancer development, gene expression data were analyzed by compiling a list of genes that were differentially expressed between each sampled tissue at each distance (0 to 20 mm) from the tumor-stromal interface, and from the tumor (0 mm) to identify expressed and biologically important genes. A paired ANOVA limma linear model (thresholds set at FDR <0.01, fold-change ≥2) was used to derive three differential gene expression lists: tumor *vs*. stroma at 5 mm; tumor *vs*. stroma at 10 mm; and tumor *vs*. stroma at 20 mm (Fig 3A). Notably, the stroma at 20 mm from tumor had nearly two-fold more differentially expressed genes than stroma at 5 mm and 10 mm, indicating that stroma at 20 mm from tumor displays the greatest difference in gene expression relative to tumor, and stroma closest to tumor displays the least, that is, the greatest tumor-like profile. Nevertheless, although stroma at 20 mm is less tumor-like, remarkably, even at this distance from tumor it continued to express a very large number of genes that are identified as tumor-like and cancer-promoting and are clearly not associated with normal breast stroma.

We next sought to determine whether a gene subset could be identified with tumor-like expression that would constitute a potential genomic ruler that defines a boundary between histopathologically identical but genomically abnormal tissue versus normal tissue. Gene expression profiles from each of these categories corresponding to tumor *vs*. 5 mm, tumor *vs*. 10 mm, and tumor *vs*. 20 mm of stroma were concatenated to obtain a non-overlapping list comprised of each of the three gene expression lists. This analysis identified 1004 genes that showed a significant change in expression between at least one distance of stroma from tumor. Within this gene expression signature, we then asked whether there are subsets of genes whose expression defines tumor as fully different from stromal tissue (*i*.*e*., a tumor signature), and a subset of genes expressed in stromal tissue at any distance that constitutes a highly tumor-like signature. To define tumor-gene and tumor-like gene expression subsets, we identified the intersection of all gene lists at each distance (Fig 3B). Genes that define the tumor as different from all stroma samples are those that are differentially expressed between stroma and tumor at each distance, comprised of those genes in the intersect of 5 mm, 10 mm and 20 mm of stroma. All other gene expression groupings that were not differentially expressed in at least one stromal axis were therefore categorized as tumor–like stromal genes. Utilizing this approach, genes were segregated into those that define a tumorigenesis process compared to housekeeping and cellular maintenance processes. Genes that define pathways involved in tumor development could then be further segmented into genes that identify tumorigenesis pathways at all distances within stroma, defined here as tumor genes, and genes that do not define tumor at all distances of stroma and therefore have shared expression between tumor and stroma, defined here as tumor-like genes (Fig 3C).

Key tumor-defining genes that encode cytokines, growth factors and other secreted factors were overexpressed in stroma and identified in the tumor-like gene list, which include well-established drivers of tumorigenesis. These include FGF2 and FGF7 that promote tumor development, cancer cell proliferation, survival and wound healing responses, as well as TGFß2, which can promote cancer progression, cancer cell invasion and metastasis. Also identified were epidermal growth factor receptor (EGFR) which is overexpressed in many cancers and stimulates a number of oncogenic signaling pathways, endomucin (EMCN), a matrix glycoprotein that interferes with cancer cell cellular adhesion, and fractalkine (CX3CL1), both of which promote cancer cell migration. We also note significantly increased expression of WISP2, a WNT-pathway protein that promotes cell invasion and metastasis, among other overexpressed mRNAs.

Interestingly, there was lower expression in the stromal tumor-like gene list of mRNAs that typically encode proteins that function to remodel the extracellular stroma to promote cell migration, invasion and survival. These include a number of matrix metalloproteinases that disrupt the extracellular matrix and activate TGF proteins, promoting cancer cell invasion and metastasis, as well as cell survival proteins such as survivin (BIRC5), which is strongly upregulated in many human cancers. We also note the lower expression of cyclin dependent kinase 1 (Cdk1) which promotes cancer cell proliferation in the stromal tumor-like compared to tumor gene list, and as well as DNA topoisomerase 2ß (TOP2A) that is overexpressed in many highly transformed cancers, including in breast cancer, in weak association with Her2.

#### Identification of gene expression transitions in stroma

To identify the distance at which stroma is most genomically dissimilar to tumor and determine whether this reflects gene expression normalization, we compared the tumor gene expression signature to the stromal tumor-like signature across each stromal distance (Fig 3D). Average tumor gene expression at each stromal distance displayed the largest differences in gene expression between tumor and stroma, highlighting that this gene signature successfully defines tumor as different from stroma. Importantly, tumor-like genes, unlike tumor-genes, displayed smaller overall gene expression differences between tumor and stroma across all stromal distances. These data indicate that the tumor enacts tumor-like gene expression in the stroma that persists at all distances into stroma and decreases only moderately even at 2 cm from tumor. Two cm was the furthest distance from tumor that could be consistently tested due to anatomical sampling considerations in the breast. In agreement with these data, when gene expression was examined for tumor-specific genes, there was a steep decline in co-expression evident between tumor and stroma, initiating at the first data point (5 mm) but thereafter maintained at all distances (Fig 3D). In contrast, expression of tumor-like genes showed a gradual reduction in expression, initiating with tumor and extending through stroma at all distances. For both tumor and tumor-like genes at 20 mm into stroma, the greatest differences between tumor and stroma were observed. This is also shown diagrammatically (Fig 3E) for all genes in the gene list for both tumor and tumor-like genes.

#### Nanostring validation of gene expression array data

**Fig 4:**
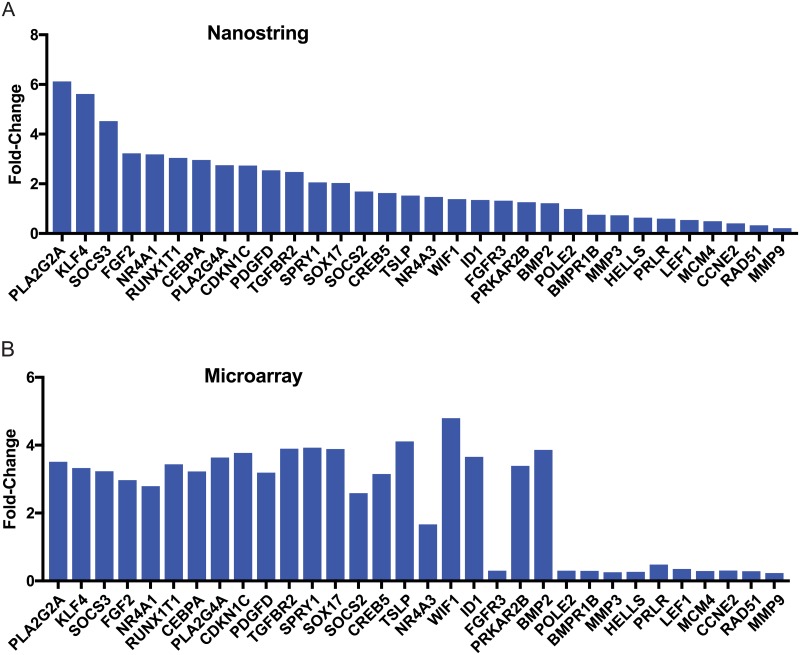
Nanostring validation of gene expression array data. (A) Thirty-two genes were selected from the gene expression profile of the 20 mm region that were in common between gene expression arrays and present on the Nanostring pan-cancer pathway gene set. Three patients were analyzed between paired tumor and stromal samples and quantified using NSolver software. (B) Quantified mRNA expression levels obtained from microarray data shown in Fig 3 for the 32 genes selected for Nanostring analysis.

To validate microarray gene expression data prior to further analysis, we selected 32 genes from the gene expression profile of the 20 mm region that were also found on the Nanostring Pan Cancer Pathway probe set. All of these genes showed either an upward or downward trend from tumor at 5 mm, 10 mm and 20 mm. Nanostring analysis was performed utilizing the pan-cancer pathway analysis gene set with 50 ng of purified RNA isolated from tissue specimens from three patients that were paired between tumor and stromal samples. Nanostring results were compared to gene expression data for each of these 32 genes (Fig 4A and 4B) and showed strong concordance in trends in gene expression between both analysis methods for stroma compared to tumor.

### A stromal tumor-like gene signature is enriched in cancer-promoting pathways throughout stroma

**Fig 5:**
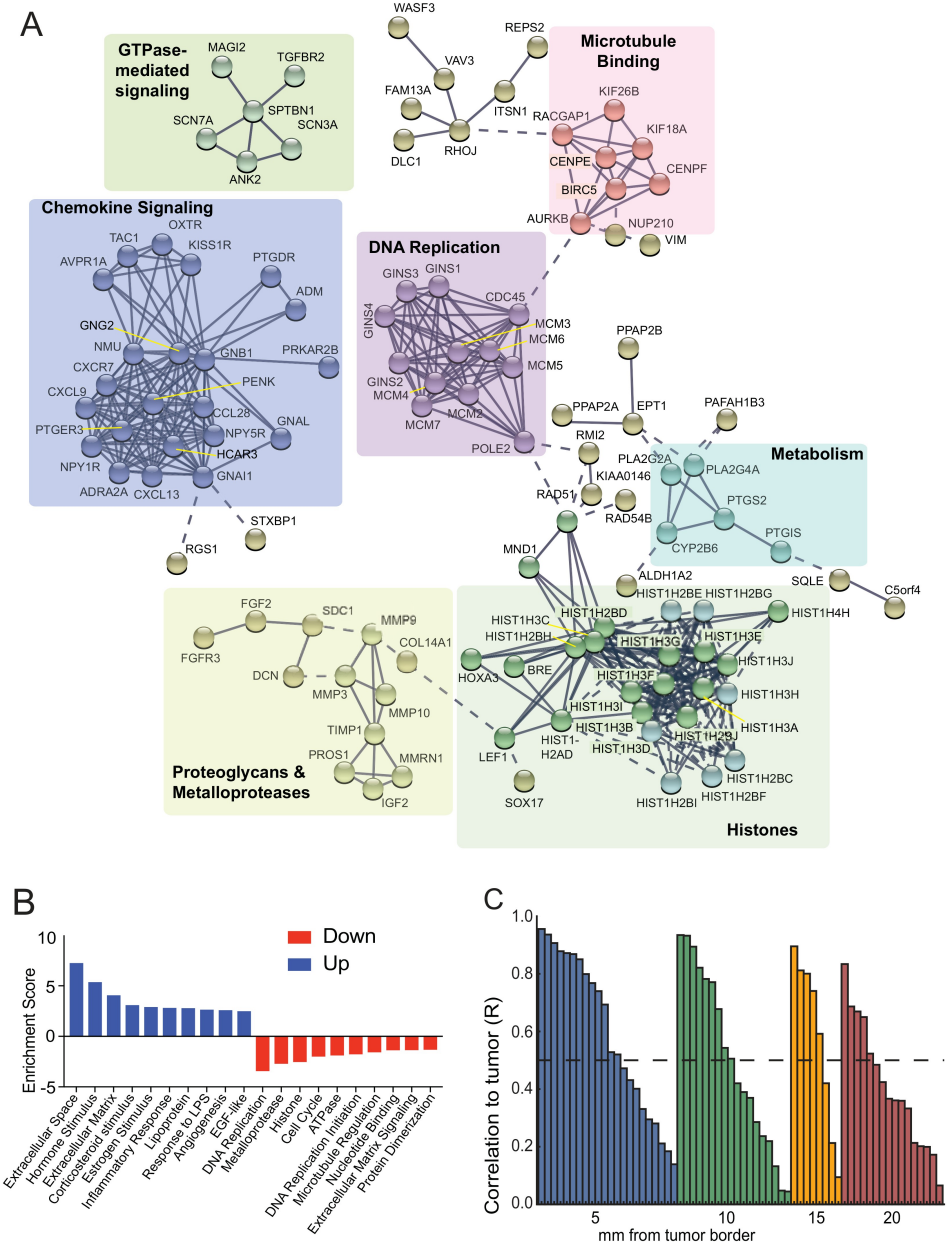
Molecular and biochemical pathway analysis of tumor-like gene expression signature. (A) STRING network analysis using a highest (0.9) confidence minimum required interaction score, showing no more than 10 interactors and removing all disconnected nodes in the network. Network clustering was done using k-means clustering. Pathway labels indicate KEGG pathway or GO Terms significantly enriched (Benjamini < 0.05) in that set of genes. (B) DAVID pathway analysis of tumor-like genes. Top ten most significant pathways in gene expression profiling that are up-regulated or down-regulated compared to tumor. (C) Pearson correlation of each patient’s tumor and stromal distance. Stromal distance R values were aggregated based on distance. Pearson correlation pertains to each patient’s tumor with all stromal tissues represented from that patient.

To further characterize genes expressed within the tumor-like signature across stromal distance, we performed pathway analysis utilizing String software and examined significant Kegg and/or GO terms (Fig 5A). We found enrichment in pathways indicating altered cell proliferation, such as DNA replication and microtubule binding, as well as pathways suggesting altered gene expression and altered cellular signaling, including histones, chemokine signaling and GTPase mediated signaling. We also found proteoglycans and metalloproteinases to be significantly enriched in the tumor-like signature indicative of stromal remodeling. To further explore whether pathways were potentially activated or inhibited in the tumor-like gene signature, we stratified tumor-like genes into up-regulated genes or down-regulated genes in the stroma compared to tumor and examined pathway enrichment in each category utilizing gene ontology software (Fig 5B). In clusters where overall stromal gene expression was significantly reduced compared to tumor, there was a reciprocal enrichment in expression of cell proliferation and invasion promoting pathways. This suggests that stromal tissue near the tumor displays selective expression of genes that promote an aggressive cancer-like phenotype, but that expression of these genes is progressively reduced the greater the distance from tumor. For instance, to breach the basement membrane, cancer cells express matrix metalloproteinases (MMPs) and alter expression of pathways related to cell adhesion [18]. As shown (Fig 3D and 3E), and captured by gene ontology analysis of our datasets (Fig 5A), there was enrichment in multiple ECM remodeling and MMP pathways in stromal tissue nearest the tumor but also more distally far into stroma. This is consistent with tumor gene expression, but in stromal cells that are histologically normal. Similarly, our analysis found that stromal regions are enriched in expression of genes for cell proliferation and cell division pathways, with the strongest expression levels closest to tumor, which then decrease compared to tumor at distal stromal sites, but also still remain significantly activated in stroma far from tumor. Interestingly, pathways seen to increase at more distal sites from tumor included those that promote chronic inflammation as well as angiogenesis (wound healing). Key unregulated immune regulatory factors were identified in Fig 3C, for instance CX3CL1, a protein that recruits leukocytes, and PLA2G4A, a protein that promotes arachidonic acid production, which incites a general inflammatory response. Pro-angiogenic factors found in stroma at the greatest distances from tumor also included HIF2α (EPAS1), which is involved in promoting angiogenesis through up-regulation of target gene VEGF-A [28] and ID1, a transcription factor that promotes maturation of endothelial progenitor cells for development of new blood vessels [29]. Though tumor cells secrete angiogenesis factors that promote tumor development and progression, stromal cells including fibroblasts and immune cells also secrete these factors that can aid in tumor angiogenesis [30].

#### The tumor-like signature in stroma distinguishes pro-neoplastic and homeostatic gene expression based on distance from tumor

The tumor-like gene profile was next used as a test set to determine whether the observed correlation in tumor-like stromal gene expression profile with distance from tumor could be validated in 17 additional patients from whom we obtained tumor and stromal samples taken at different distances from the tumor boundary. Correlating each patient’s tumor with stromal distance, we found that biopsy specimens 20 mm from the tumor-stromal interface had the lowest correlation with tumor gene expression compared to specimens closer to tumor, as observed in the test set. Specifically, only 31% of specimens at 20 mm from tumor had an R value >0.5, compared to 63% at 15 mm, 50% at 10 mm, and 59% at 5 mm (Fig 5C). Therefore, the tumor-like gene expression profile became progressively less tumor-like with distance from tumor, but at 20 mm, there was a sharper decrease in tumor-like gene expression observed independent of tumor size and grade. These data indicate that the stromal region furthest from the tumor expresses the largest heterogeneity of genes. We therefore sought to better define the types of genes altered in expression and the least like tumor in the 20 mm stromal region.

#### Genomic composition of the most distal stromal region from tumor

**Fig 6:**
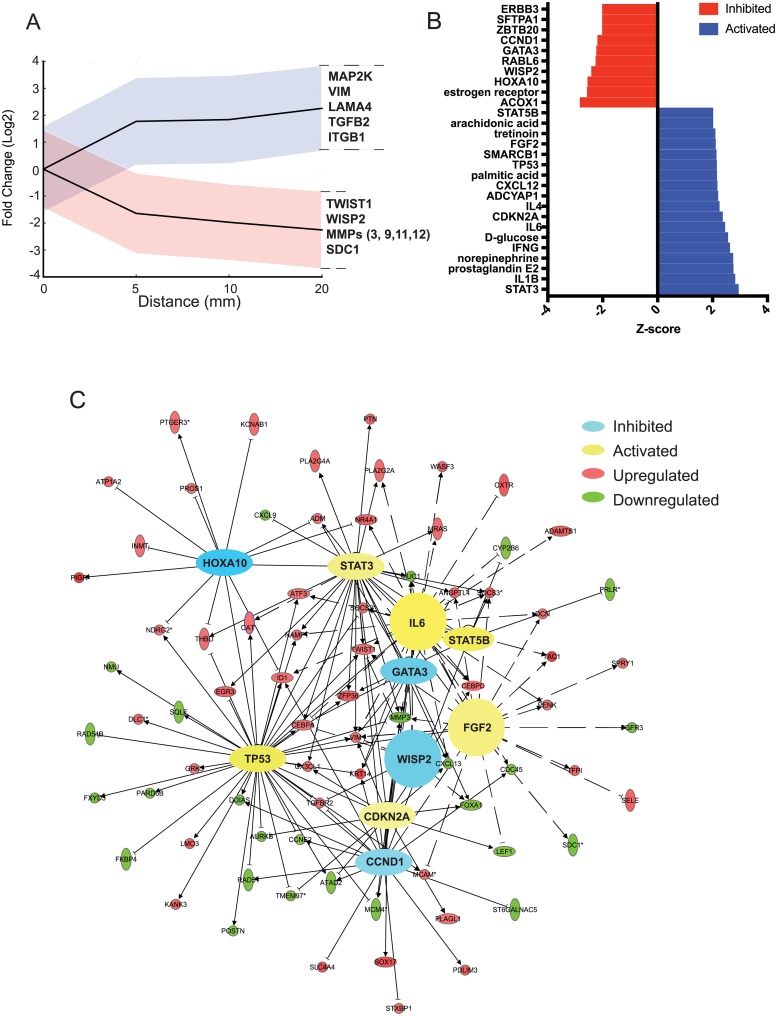
Gene expression signature of the uniquely differentially expressed genes between 20 mm stromal region *vs*. tumor (0 mm). (A) Average fold-change of 20 mm stromal region gene expression across each stromal distance. Blue is indicative of the standard deviation of the fold-change at each distance for genes upregulated across stroma. Red indicates the standard deviation of fold-change for genes at each distance that are downregulated across stroma. (B) Pathways predicted to be activated or inhibited in the 20 mm stromal region using IPA upstream regulator analysis software. Pathways with unbiased significant p-values were selected. (C) IPA network analysis highlighting upstream regulator pathways that are related to homeostasis, tumor inhibiting, and tumor promoting activities. Pathways are highlighted in orange (activated) and blue (inhibited) based on predicted z-score. Genes found to be upregulated (red) or downregulated (green) in the 20 mm region are clustered based on pathway interaction.

The gene expression profile of the 20 mm stromal region was compared to all stromal distances and to tumor (Fig 6A). While the expression of genes was significantly different between tumor and the most distant stromal region (20 mm), upstream pathway analysis indicated that the distant stromal region is still in a state of pro-neoplastic gene expression, and yet is not transformed by any histopathological criteria (Fig 6B). Rather, the stromal region distant from tumor is in a state of genomic flux. In addition to activation of pro-neoplastic pathways, we also observed significant expression of genes involved in homeostatic cell-normalization pathways, including tumor suppressor gene activities. Among the expressed pro-transforming genes and pathways, we note those involved in cell activation processes, cell proliferation, angiogenesis, cell migration, ECM remodeling, FGF expression, oncogenic signaling and the EMT. Specifically, GATA3 signaling, a regulator of breast epithelial differentiation was predicted to be inhibited, indicative of stroma that is in a more stem cell-like state [31]. Also noted are factors consistent with a pro-inflammatory state associated with tumor promotion, including the up-regulation of IL-1ß which can recruit tissue remodeling leukocytes [32]. The most tumor distal stromal region likely also has increased metabolic activity, shown by the strong up-regulation in acetyl CoA, which may reflect a higher rate of metabolism to support accelerated cell proliferation rate and activities of cancer cells [6, 16]. We also note the up-regulation of MAP2K expression, possibly through activation of FGF2 genes, which can promote cell migration [33] (Fig 5C).

Among the expressed pro-normalization genes are those involved in down-regulation of WNT and the WNT pathway as a result of WIF1 up-regulation of TP53, TWIST-1, WISP2, certain MMPs [3, 11, 34] and syndecan 1 (SDC1) [35]. SDC1 is associated with stromal and vascular proliferation and can be a marker of normal tissue. However, SDC1 can also be found on carcinoma-associated fibroblasts (CAFs) and promote tumor progression [36]. Perhaps due to suppression of the Wnt pathway, cyclin dependent kinase D1 (CCND1) activity, a transcriptional target of Wnt signaling, was predicted to be decreased in the 20 mm region. CCND1 has been shown to be amplified in a large number of breast tumors and as a key regulator of the G1/S cell cycle transition, promotes tumor cell proliferation and metastasis [37]. Low CCND1 pathway activation highlights the renormalization of proliferation pathways. Though decreased CCND1 activity may block proliferation, low CCND1 has been shown to correlate with high ID1 levels, as seen in the 20 mm region, which may promote more aggressive breast cancer tumor phenotype and metastasis [38]. Collectively, these genes inhibit MMP activity and cellular apoptosis, increase cellular adhesion and diapedesis compared to stromal regions closer to tumor. Factors such as VIM, ITGß1-BP1 and MAP2K were also increased in expression in the distal region of stroma from tumor and can contribute to tumor dormancy [18]. The activation of STAT3, STAT5B, and CDKN2A promotes a state of equilibrium and has tumor suppressive activities. Both STAT3 and STAT5B are involved in breast cell growth and survival. When activated, STAT5B decreases proliferation and fosters apoptosis [39]. CDKN2A regulates the cell cycle by producing p16^ink4a^ that halts proliferation, protects p53 and permits tumor suppression. However, in breast cancer, IL-6 promotes the activities of STAT3. This leads to invasion through TWIST-1 by increasing motility and the EMT [40].

Finally, there were a surprisingly small number of upregulated pro-tumorigenic immune factor genes in the distal 20 mm region of the stroma. Identified were TGFß2, a primary mediator of TGFß, which can be either tumor suppressing or tumor promoting, and which may reflect immune equilibrium [41]. Laminin-4 (LAMA4), which is part of the basement membrane that promotes cell adhesion [42]. These pathways therefore represent both homeostatic and pro-transforming activities where immune factors, angiogenesis and cell morphology dominate.

## Discussion

It has long been debated whether excising larger regions of non-cancerous tissue (the surgical margin) for invasive breast actually impacts on the rate of ipsi-lateral breast cancer recurrence [43]. Consensus guidelines regarding the adequate surgical margin in breast and other surgeries are based solely on histopathologic parameters and do not integrate information regarding the activation state of, or gene expression in the surrounding stroma [34, 44]. There are no surgical or treatment guidelines that include parameters of the tumor microenvironment.

The stroma surrounding tumors can express genes that promote angiogenesis, cellular invasion, cell growth, immune evasion and increased energy metabolism, among other pro-neoplastic pathways [16, 45]. It has been suggested that targeting stroma cancer promoting pathways may provide a more effective strategy to prevent cancer recurrence [16]. In effect, that is what neoadjuvant radiation therapy likely accomplishes, at least in part, but without consideration of the state of stromal neoplastic activation.

While the EMT promotes cancer metastasis through increased cancer cell invasion, drug resistance, loss of cell–cell adhesion, and tumor recurrence [33, 42], ECM genes have also been shown to be activated in the corresponding breast tumor stroma that support the aggressive EMT phenotype [22], such as secretion of MMPs that promote invasion by cancer associated fibroblasts (CAFS) [6, 33]. Our study found a negative correlation in gene expression compared to tumor distance for ECM remodeling and metalloproteinase gene expression. This suggests that expression of ECM remodeling genes that can breach basement membrane and facilitate cancer cell invasion become weaker at distances further from the tumor, but still retain expression far distal from the tumor in the morphologically normal breast. We also provide evidence for other key processes in stromal microenvironment remolding that promote cancer progression and metastasis, including formation of the metastatic niche, metabolic stimulation, immune modulation, stimulation of tumor cell migration and angiogenesis.

Our study included all post-tumor stromal cells in the analysis. Of these cells, CAFs are the most abundant cell type in the tumor-free stroma, many of which express genes activated in response to tumor-related immune infiltration, cytokines and other signals [46, 47]. CAFs can promote tumor proliferation, invasion, angiogenesis, tissue remodeling and presumably recurrence [48, 49]. However, there are no truly specific markers for CAFs, other than expression of myoblast protein alpha smooth muscle actin alpha SMA), which itself is quite variable [47]. We therefore did not stain for the population of CAFs, which no doubt are included in our analysis of the post-tumor stroma. Given the role of CAFs in preventing anti-tumor CD8^+^ cytotoxic T cell responses [49, 50], and pro-oncogenic gene expression profiles we observed deep within the tumor-free stroma, which includes immune-related gene expression, our study likely measured both CAF and non-CAF stromal cell gene expression.

Tumor and stromal heterogeneity have been linked to response and resistance to chemotherapy [14, 16, 44, 51]. Stromal gene signatures have been found to predict chemotherapy resistance, which includes processes related to ECM remodeling, angiogenesis, and cell proliferation [8, 14] as found here. Stromal signatures have also found to be predictive of neoadjuvant therapy response [14]. Similarly, radiation therapy has an impact on the both tumor and stroma, shown clearly by the NSABP B-06 trial which established the added benefit of whole breast irradiation in reducing recurrence when combined with breast conserving surgery [2]. On a molecular level, radiation therapy may deactivate the reactive stroma or induce apoptosis in activated, pro-neoplastic stromal cells, thereby minimizing cancer-promoting features of the cellular environment [52].

A better understanding of interactions between tumor and the surrounding stroma could lead to improved treatment markers, risk assessment and a greater impact on cancer therapy [6, 53]. Functional analysis of tumor-like genes suggests that pathways shared between tumor and stroma promote malignancy. In this regard, it is surprising the extent to which we found pro-malignant, tumor-like gene expression in cancer-devoid, histopathologically normal tissue surrounding the tumor. Mechanistically, there is currently little understanding by which establishment of tumor-related gene expression can occur deep within stroma. Tumor-like gene expression in deep stroma was not related to a desmoplastic response since few genes associated with that type of response were found to be upregulated. Our study provides genomic evidence within the cancer-free stroma for such long-range transforming gene expression responses and shows that there is a quantifiable gene expression relationship to tumor that can support carcinogenesis. Unfortunately, the sample size of our study was not large enough to allow statistically significant analysis of correlations between particular tumor and patient characteristics with gene expression data. Nevertheless, our findings also provide the possibility of more accurately and individually describing risk and treatment outcome, to be developed in larger studies.

## Supporting information

S1 Table Clinical and pathological characteristics of patient specimens. (PDF)

S2 Table Patient characteristics. (PDF)

S1 File Tumor and tumor-like stromal signatures. Genome-wide mRNA analysis derived from filtered datasets of S2–S5 Files comparing tumor and tumor-like gene expression profiles.
(XLSX)

S2 File Down_tumor_like_clusters. Genome-wide mRNA analysis of downregulated stromal genes based on tumor-like profiles.
(XLSX)

S3 File Tumor_down_stroma_up_DAVID. Genome-wide mRNA analysis of downregulated tumor-like genes in stromal profiles and upregulated stromal gene profiles analyzed by DAVID.
(XLSX)

S4 File Tumorup_stromadown_DAVID. Genome-wide mRNA analysis of upregulated tumor-like genes in stromal profiles and downregulated stromal gene profiles analyzed by DAVID.
(XLSX)

S5 File Up_tumor_like_clusters. Genome-wide mRNA analysis of upregulated tumor-like genes in stromal profiles.
(XLSX)
