# Supplementary material for: A genomic ruler to assess oncogenic transition between breast tumor and stroma
Source: PLoS One. 2018 Oct 16;13(10):e0205602. doi: 10.1371/journal.pone.0205602 (PMC6191134; doi:10.1371/journal.pone.0205602)
Supplement: S1 Table — (PDF) [file pone.0205602.s001.pdf]

S1 Table. Clinical and pathological characteristics of patient specimens

Clinical-Pathologic Characteristics of Patient Samples

Patient ID	Specimen distance from tumor-free margin (mm)					Grade	HR Status	HER2	Stage	Breast Density	Age	Race	Lymph Node Status
	0	5	10	15	20								
**1**		X	X	X	X	PD	-	-	IIB	1	80	White	+
**1100**	X	X				MD	-	+	IIA	2	55	Black	+
**1105**	X	X				PD	+	+	IIIA	2	81	Hispanic	+
**1106**			X	X		PD	-	-	I	3	39	Asian	+
**1108**	X	X	X	X	X	PD	+	-	I	2	61	Black	+
**1120**	X		X	X	X	PD	+	-	IIA	2	59	White	+
**1121**	X	X	X	X		PD	-	-	I	3	36	White	-
**1122**	X	X				WD	+	-	IIA	1	67	White	+
**1123**	X		X	X		PD	+	-	IIA	2	41	White	+
**1124**	X	X		X	X	WD	+	-	I	2	46	White	+
**1127**	X	X	X		X	PD	+	+	IIB	3	57	White	+
**1130**					X	PD	+	-	IIIC	2	55	Asian	+
**1131**	X	X	X			PD	+	+	IIA	4	41	White	-
**1132**	X	X	X		X	PD	+	+	IIA	2	35	Hispanic	+
**1136**	X	X	X		X	PD	-	+	IIA	3	55	Black	-

S1 Table. Clinical and pathological characteristics of patient specimens

**1140**	X	X	X			MD	+	-	I	3	41	Asian	-
**1141**	X	X	X		X	PD	+	+	IIA	2	42	White	-
**12**	X	X				PD	+	+	IIIA	2	55	Black	-
**120T**	X					MD	+	+	IIIA	3	41	Black	-
**147**	X	X	X		X	PD	+	-	IIA	2	68	Hispanic	+
**2**	X	X				MD	+	-	IIA	4	39	Asian	-
**3**	X		X	X	X	MD	+	-	I	4	39	Hispanic	-
**4**	X	X		X	X	MD	+	-	IIB	3	43	Black	+
**5**	X	X	X		X	MD	+	+	IIIB	3	50	Asian	+
**6**		X		X	X	MD	-	+	IIA	3	42	White	-
**7**	X	X	X			MD	+	-	I	1	41	Black	-
**8**	X	X	X	X	X	MD	+	-	IIA	2	51	Hispanic	-
**B1**	X	X	X		X	PD	+	+	IIA	2	50	Asian	-
**B2T**	X					MD	-	-	IIIC	3	60	Asian	+
**B5**		X	X		X	PD	-	-	IIIA	3	49	Asian	+
**B6**	X	X	X		X	MD	+	-	II	3	60	Asian	-
**B7**	X				X	MD	+	-	IIA	3	69	Asian	-
**B8**	X	X	X		X	PD	+	-	I	2	62	Asian	-

*Abbreviations: Poorly differentiated (PD), moderately differentiated (MD), well differentiated (WD), positive (+), negative (-), HR (hormone receptor)
