# Supplementary material for: A genomic ruler to assess oncogenic transition between breast tumor and stroma
Source: PLoS One. 2018 Oct 16;13(10):e0205602. doi: 10.1371/journal.pone.0205602 (PMC6191134; doi:10.1371/journal.pone.0205602)
Supplement: S2 Table — (PDF) [file pone.0205602.s002.pdf]

S2 Table. Patient characteristics

Condition		# of samples	% of samples
**Grade**	MD	13	39.3939394
	PD	18	54.5454545
	WD	2	6.06060606
**Receptor**	Positive	25	75.7575758
	Negative	8	24.2424242
	HER2	12	36.3636364
**ln status**	Positive	17	51.5151515
	Negative	16	48.4848485
**Ethnicity/race**	Asian	11	33.3333333
	Black	7	21.2121212
	Hispanic	5	15.1515152
	White	10	30.3030303
